# Metabolomic Alteration of Oral Keratinocytes and Fibroblasts in Hypoxia

**DOI:** 10.3390/jcm10061156

**Published:** 2021-03-10

**Authors:** Hiroko Kato, Masahiro Sugimoto, Ayame Enomoto, Miku Kaneko, Yuko Hara, Naoaki Saito, Aki Shiomi, Hisashi Ohnuki, Kenji Izumi

**Affiliations:** 1 Research Center for Advanced Oral Science, School of Medical and Dental Sciences, Niigata University, Nii-gata 951-8514, Japan; 2 Division of Biomimetics, School of Medical and Dental Sciences, Niigata University, Niigata 951-8514, Japan; hyuko@dent.niigata-u.ac.jp (Y.H.); nao-saito@dent.niigata-u.ac.jp (N.S.); oonuki.hisashi@maroon.plala.or.jp (H.O.); 3 Laboratory of Advanced Cosmetic Science, School of Pharmaceutical Sciences, Osaka University, Suita, Osaka 565-0871, Japan; 4 Research and Development Center for Minimally Invasive Therapies, Health Promotion and Preemptive Medicine, Tokyo Medical University, Tokyo 160-8402, Japan; mshrsgmt@tokyo-med.ac.jp; 5 Institute for Advanced Biosciences, Keio University, Tsuruoka, Yamagata 997-0052, Japan; ayame.e@ttck.keio.ac.jp (A.E.); kkk-miku@ttck.keio.ac.jp (M.K.); 6 Division of Dental Education Research Development, School of Medical and Dental Sciences, Niigata University, Niigata 951-8514, Japan; as.aki.mh@gmail.com

**Keywords:** oxygen biology, metabolomics, oral keratinocytes, oral fibroblasts

## Abstract

The oxygen concentration in normal human tissue under physiologic conditions is lower than the atmospheric oxygen concentration. The more hypoxic condition has been observed in the cells with wound healing and cancer. Somatic stem cells reside in a hypoxic microenvironment in vivo and prefer hypoxic culture conditions in vitro. Oral mucosa contains tissue-specific stem cells, which is an excellent tissue source for regenerative medicine. For clinical usage, maintaining the stem cell in cultured cells is important. We previously reported that hypoxic culture conditions maintained primary oral keratinocytes in an undifferentiated and quiescent state and enhanced their clonogenicity. However, the metabolic mechanism of these cells is unclear. Stem cell biological and pathological findings have shown that metabolic reprogramming is important in hypoxic culture conditions, but there has been no report on oral mucosal keratinocytes and fibroblasts. Herein, we conducted metabolomic analyses of oral mucosal keratinocytes and fibroblasts under hypoxic conditions. Hypoxic oral keratinocytes and fibroblasts showed a drastic change of metabolite concentrations in urea cycle metabolites and polyamine pathways. The changes of metabolic profiles in glycolysis and the pentose phosphate pathway under hypoxic conditions in the oral keratinocytes were consistent with those of other somatic stem cells. The metabolic profiles in oral fibroblasts showed only little changes in any pathway under hypoxia except for a significant increase in the antioxidant 2-oxoglutaric acid. This report firstly provides the holistic changes of various metabolic pathways of hypoxic cultured oral keratinocytes and fibroblasts.

## 1. Introduction

Oxygen plays an important role in energy metabolism and signal transduction in maintaining the homeostasis of the microenvironment of the living body. The response of cultured cells to oxygen concentration has attracted attention as a physiological factor. The partial pressure of oxygen (pO_2_) varies throughout the body ranging from 9% pO_2_ in the lungs to 0.1% pO_2_ in the peripheral tissues, depending on blood flow. Mohyeldin et al. [1] reported that the oxygen concentration is 7% pO_2_ in the dermis and 0.2–8% pO_2_ in the epidermis. Skin appendages show lower oxygen concentrations than the epidermis; hair follicles have 0.1–0.8% pO_2_ and sebaceous glands have 0.1–1.3% pO_2_ [2,3]. Culturing keratinocytes at 2% oxygen concentration has been reported to suppress stratification, cellular enlargement, and differentiation [4]. In cultured fibroblasts, the production levels of vascular endothelial growth factor (VEGF) and type I collagen, which are related to angiogenesis, collagen production, and tissue remodeling, have been altered in hypoxic conditions, indicating their roles in tissue remodeling during wound healing [5]. Hypoxic conditions increase the induction efficiency of induced pluripotent stem (iPS) cells and promote the undifferentiated cell proliferation of mesenchymal stem cells and neural stem cells [6,7,8]; therefore, hypoxic cultures have been attempted to be applied in regenerative medicine.

Hypoxia-inducible factor 1-alpha (HIF-1a) is the main regulator of cellular hypoxic response and binds to promoters of genes encoding glucose transporters and glycolytic enzymes that are important for metabolic reprogramming from oxidative phosphorylation to glucose metabolism [3]. Decreased oxygen consumption in the mitochondria prevents the production of reactive oxygen species (ROS) by suppressing the electron transport system, resulting in cell survival under hypoxia [9]. In cancer tissues, energy production dominantly depends on the glycolysis pathway (Warburg Effect) [10], and various metabolic shifts, such as glutaminolysis activation, due to insufficient nutrition and oxygen have been observed [10].

Oral mucosa-derived cells are a useful source for regenerative medicine and basic research, including disease model fabrication [11]. The influence of oxygen concentrations on cancer and wound healing in oral mucosa has been examined [12,13]; however, no report on the oxygen concentration in vivo has been published. Since the culture of oral mucosal epithelial cells under hypoxic conditions suppresses differentiation and senescence and promotes colony formation, hypoxic exposure is beneficial for applications in regenerative medicine, similar to other cells [14].

Although metabolic reprogramming plays an important role in stem cell biology and pathological conditions under hypoxia, the effects of hypoxia on oral mucosal keratinocytes and fibroblasts are poorly elucidated. Therefore, the purpose of this study is to demonstrate the characteristics of the metabolic mechanisms of oral mucosal keratinocytes and fibroblasts in hypoxia.

## 2. Materials and Methods

### 2.1. Primary Cell Culture under Hypoxic Conditions

Primary human oral keratinocytes were isolated and cultured routinely in EpiLife^®^ medium (Thermo Fisher Scientific, Waltham, MA, USA) supplemented with EpiLife^®^ Defined Growth Supplements (Thermo Fisher Scientific), 0.06 mM Ca^2+^, gentamicin (5.0 μg/mL; Thermo Fisher Scientific), and amphotericin B (0.375 μg/mL; Thermo Fisher Scientific) as described previously [15].

Primary oral fibroblast cultures were established by an explant culture technique using the connected tissue after the epithelial layer was scraped off. Small explants were placed in a 60-mm Petri dish (Corning, New York, NY, USA) and incubated in Dulbecco’s modified Eagle medium (DMEM; FUJIFILM Wako Pure Chemical Corporation, Osaka, Japan) supplemented with 10% fetal bovine serum (Thermo Fisher Scientific), gentamicin, and amphotericin B as previously described [16].

Six of the oral mucosa keratinocytes and fibroblasts used in the study were from passages 3 to 5. To culture the cells under hypoxic conditions, culture vessels were placed in a humidified modular incubator chamber (Billups Rothenberg, Inc., Del Mar, CA, USA), flushed for 2 min with a gas mixture of either 2.0% O_2_ (5.0% CO_2_–93.0% N_2_) or 0.5% O_2_ (5.0% CO_2_–94.5% N_2_), and then placed in an incubator at 37°C. Cells were fed with either a 2% or a 0.5% O_2_ tension equilibrating complete media every other day. As a normoxic condition (20% O_2_), vessels were placed in ambient oxygen in an incubator at 37 °C with a humidified 5.0% CO_2_ environment as described previously [17].

### 2.2. Metabolite Extraction

Oral keratinocytes and fibroblasts were plated on 100-mm dishes and cultured under 2% or 0.5% O_2_ conditions for 24 h or 72 h. Each experimental condition was performed in duplicate for metabolite extraction and for cell counting after trypsinization to normalize the metabolomics data. For metabolite extraction, cells were washed twice with 5 mL of ice-cold 5% D-mannitol and then immersed in 1 mL of methanol containing internal standards (25 mM each of methionine sulfone, 2-[N-morpholino]-ethanesulfonic acid, and D-camphor-10-sulfonic acid) for 10 min on ice. The lysate was scraped and collected in 1.5 mL tubes, snap-frozen by liquid nitrogen, and then stored at −80 °C until analysis. To 400 µL of the dissolved samples, 400 µL of chloroform and 200 µL of Milli-Q water were added, and the mixture was centrifuged at 10,000× *g* for 3 min at 4 °C. The aqueous layer was filtered to remove large molecules by centrifugation through a 5-kDa cut-off filter (Merck Millipore, Burlington, MA, USA) at 9100× *g* for 2.0 h at 4 °C. Then, 320 µL of the filtrate was concentrated by centrifugation and dissolved in 50 µL of Milli-Q water containing reference compounds (200 µM each of 3-aminopyrrolidine and trimesate) immediately before the capillary electrophoresis time-of-flight mass spectrometry (CE-TOF-MS) analysis.

The instrumentation and measurement conditions used for CE-TOF-MS were described elsewhere [18]. Briefly, cations and anions in the 50–1000 *m*/*z* range were analyzed independently. The migration times of each chromatograph were normalized by dynamic programming-based methods, and metabolite identification was conducted by matching the *m*/*z* and corrected migration times with our standard library [19]. To calculate the absolute concentration of each metabolite, corresponding standard compounds were prepared. The peak area of each metabolite was divided by those of the internal standard compound (methionine sulfone) to calculate the relative area by eliminating the unexpected bias of MS sensitivity fluctuation. The standard mixture was measured before the sample measurement, and based on the ratio of relative areas of each metabolite in the sample and standard mixture, the concentrations were calculated. The upper and lower quantifications limits were already measured using standard mixtures, and the peaks under lower limits were treated as not detected (N.D.).

The overall metabolomic profiles were assessed by clustering analysis. Statistical analysis was performed by a two-tailed Student’s *t*-test. Differences were considered significant at *p* 0.05. The data processing and pathway visualization were conducted by our proprietary software, MasterHands and Pathway Visualization [19,20,21].

Frequently observed peaks (≥85% of all samples) were used for heatmap visualization and PCA. Pearson correlation was used for the metabolite alignment at heatmaps. These analysis and data visualizations were conducted using MetaboAnalyst (ver 5.0, https://www.metaboanalyst.ca/, acceded on 21 January 2021), GraphPad Prism (version 5.04, GraphPad Software, San Diego, CA, USA), and MeV TM4 software (version 4.9.0, http://mev.tm4.org/, acceded on 20 January 2021).

## 3. Results

### 3.1. The Metabolomic Concentration of Oral Keratinocytes and Oral Fibroblasts

**Figure 1:**
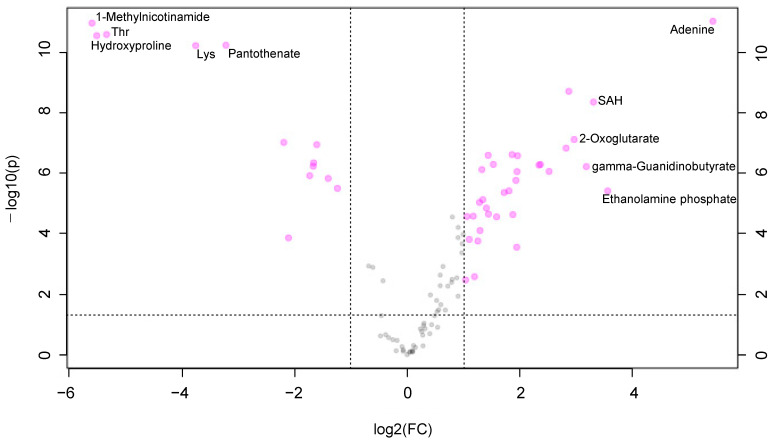
Volcano plots of the metabolomic profile. X-axis indicates the log_2_(fold change (F.C.)) of oral keratinocytes (*n* = 7)/fibroblasts cultured (*n* = 7) in a 20% oxygen environment. The Y-axis indicates –log_10_(P) using the *t*-test. Before the calculation of F.C., each metabolite concentration was divided by the average concentration of each sample to eliminate sample-dependent bias. Each plot indicates a metabolite. The metabolites showing a large difference in F.C. (log_2_(F.C.) −1 or 1 log_2_(F.C.)) and small *p*-value (*p* 0.05) were colored in pink.

Metabolomic analysis successfully identified and quantified 208 and 203 metabolites in cultured oral keratinocytes and fibroblasts, respectively, under atmospheric oxygen concentration. The log_2_ of fold change (F.C.) in the metabolite concentration between these cells and *p*-values are shown in the scatter plot (Figure 1). The 33 and 13 metabolites showed higher (log_2_ F.C. 1) and lower (log_2_ F.C. −1) concentration of keratinocytes at a significant level (*p* 0.05), respectively.

**Figure 2:**
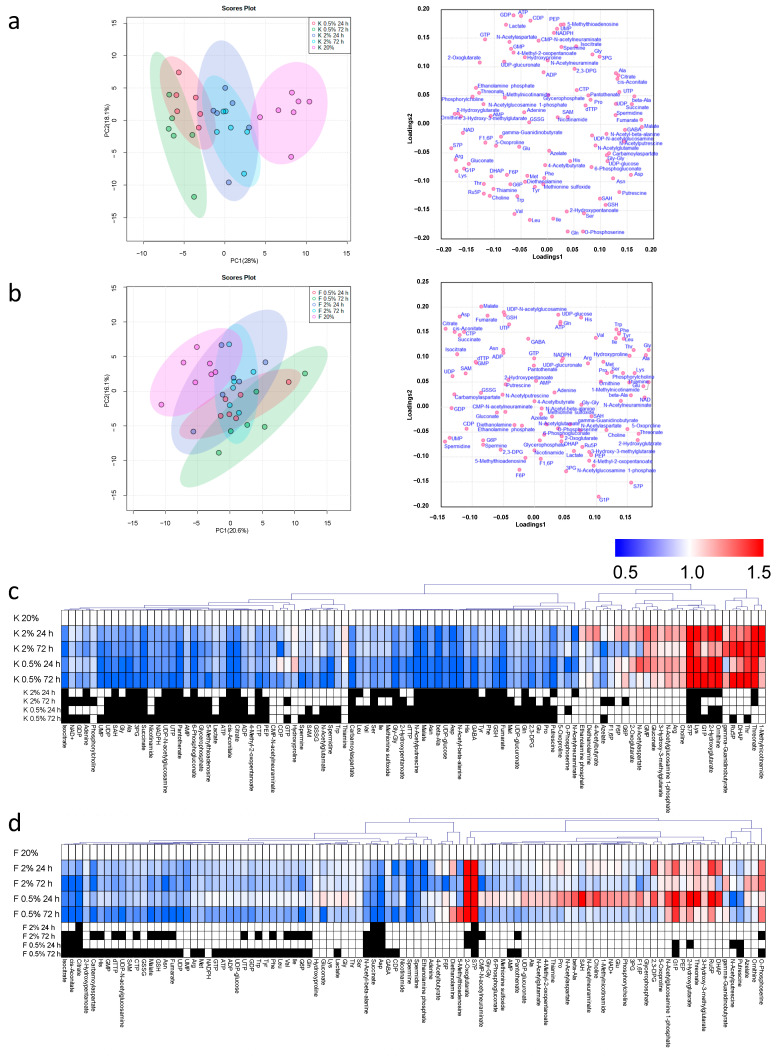
Principal component analyses (PC) of keratinocytes (**a**) and fibroblasts (**b**). The left and right panels were score plots and loading plots, respectively. The X and Y-axes indicated the first and the second PC with contribution ratios. Each plot indicates sample and metabolites in score and loading plots, respectively. Each metabolite concentration was divided by the sum of all metabolite concentrations of each sample, transformed to log_2_ and Z-score before PC analysis. Heatmap of keratinocytes (**c**) and fibroblasts (**d**). Each box in the heatmap indicated the fold change (F.C.); i.e., the averaged values of metabolite concentrations of each sample were divided by those of the reference samples cultured under 20% O_2_. The color bar indicated F.C. The black boxes indicated *p* 0.05 (Student’s t-test) between each sample and the reference sample. The cells were cultured under the condition with 20% O_2_ (*n* = 7), 2% O_2_ for 24 h (*n* = 6), 2% O_2_ for 72 h (*n* = 6), 0.5% O_2_ for 24 h (*n* = 6), and 0.5% O_2_ for 72 h (*n* = 6).

PCA was performed individually for each cell type to evaluate the effect of oxygen concentrations. The score plots of keratinocyte revealed that the samples with normoxic conditions were located at the right (labeled as K 20%), 2% O_2_ conditions were located at the center (K 2% 24 h and 72 h), and 0.5% O_2_ conditions were located at the left (K 0.5% 24 h and 72 h) (Figure 2a). Thus, the first principal component (PC1) reflects the oxygen condition. The loading plots showed the distribution of various metabolites along with PC1. Among amino acids, arginine (Arg), lysine (Lys), and threonine (Thr) showed a relatively small PC1 value (PC1 −0.1), whereas the aspartate (Asp), asparagine (Asn), and alanine (Ala) showed larger PC1 values (PC 0.15). Intermediate metabolites at tricarboxylic acid (TCA) cycles, such as citrate, succinate, fumarate, malate, and cis-aconitate also showed larger PC values (PC 0.1).

The score plots of fibroblast revealed that the samples with normoxic conditions (F 20%) were located at the upper left, the samples in 0.5% O_2_ conditions for 72 h (F 0.5% 72 h) were located at the lower right vice versa, and the samples with other conditions were located between them (Figure 2b). Both PC1 and the second PC (PC2) reflect the difference in the oxygen used. The intermediate metabolites in glycolysis, such as glucose 1-phosphate (G1P), 3-phosphoglyceric acid (3PG), and phosphoenolpyruvate (PEP) are located at the lower right area. Oppositely, organic acids, such as succinate, isocitrate, malate, and fumarate were located in the upper left area.

**Figure 3:**
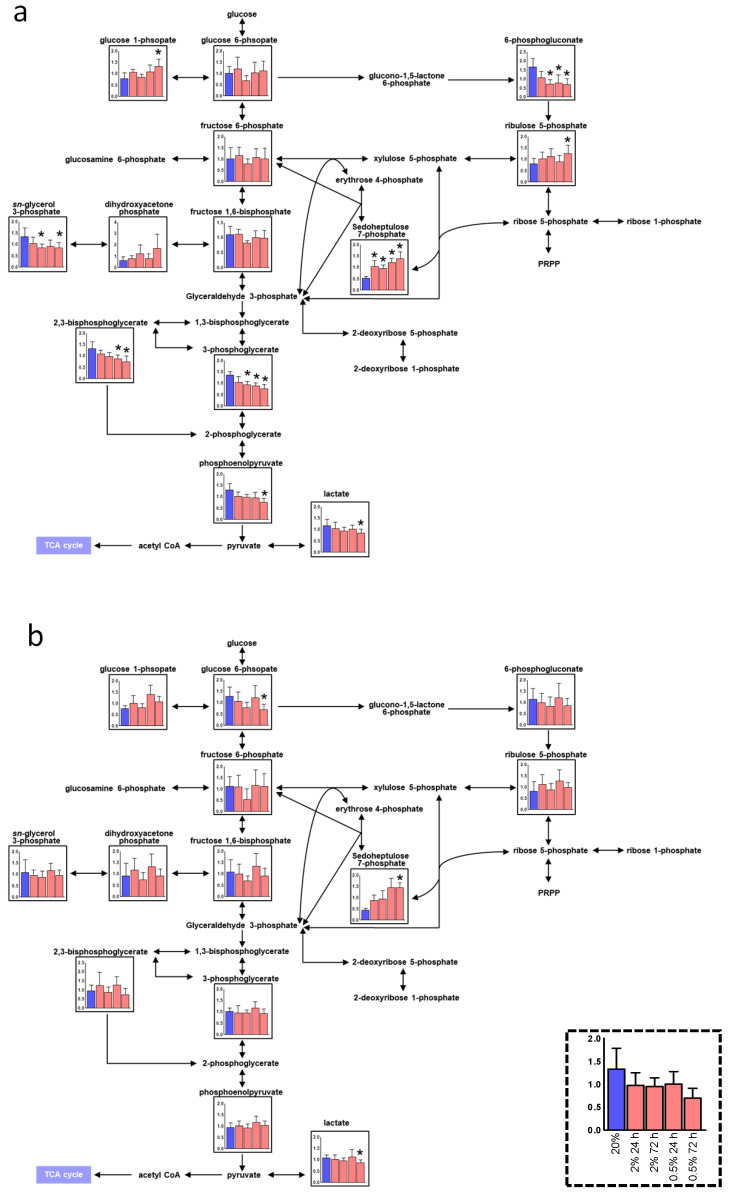
Metabolic pathways of glycolysis and pentose phosphate pathway (PPP) of oral keratinocytes (**a**) and oral fibroblasts (**b**) in hypoxic culture (*n* = 6). Quantified metabolite concentrations are shown as bars; ambient oxygen cultured (blue), 2% O_2_ for 24 h, 2% O_2_ for 72 h, 0.5% O_2_ for 24 h, and 0.5% O_2_ for 72 h from the left, respectively. *p*-values were calculated using Student’s *t*-test (two-tailed, unequal variance). * *p* 0.05 was shown for comparison between each data with data for 20% oxygen.

The heatmaps of keratinocytes (Figure 2c) and fibroblasts (Figure 2d) show the F.C. of metabolite concentrations of each sample divided by those of the reference sample under atmospheric oxygen concentration, i.e., the red and blue colors indicate the relatively higher and lower concentration compared to the ones in the reference sample. Overall, the heatmap of keratinocytes showed clearer red and blue colors compared to one of the fibroblasts. Therefore, the metabolomic impact depending on the O_2_ condition and culture duration of keratinocytes was more significant compared to the fibroblasts. In both samples, the number of metabolites showing higher concentration was fewer than the number of metabolites showing lower concentration. Only sedoheptulose 7-phosphate (S7P), an intermediate metabolite in the pentose phosphate pathway (PPP), showed significantly higher (*p* 0.05) compared to the reference samples in any culture condition in both samples (Figure 2c,d and Figure 3).

### 3.2. Alteration of the Pentose Phosphate Pathway (PPP) and Urea Cycle under Hypoxia

**Figure 4:**
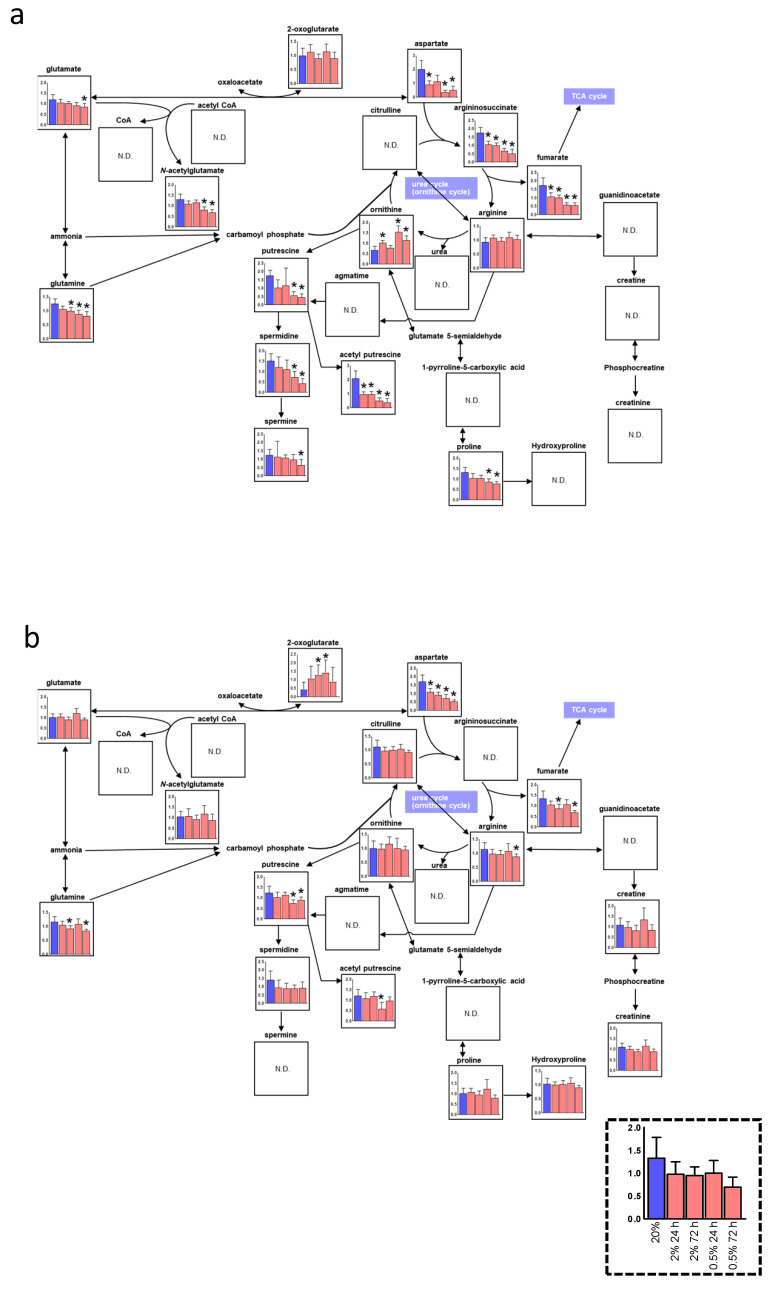
Metabolic pathway maps of the urea cycle of oral keratinocytes (**a**) and oral fibroblasts (**b**) in hypoxic culture (*n* = 6). Quantified metabolite concentrations are shown as bars; ambient oxygen cultured (blue), 2% O_2_ for 24 h, 2% O_2_ for 72 h, 0.5% O_2_ for 24 h, and 0.5% O_2_ for 72 h, from left, respectively. *p*-values were calculated using Student’s *t*-test (two-tailed, unequal variance). * *p* 0.05 was shown for comparison between each data for 20% oxygen.

Among various metabolic pathways, the PPP and urea cycle with various ambient oxygen cultures showed a distinct difference between oral keratinocytes and fibroblasts (Figure 3 and Figure 4). Among the 13 metabolites in the PPP, 11 and three showed significant differences depending on the O_2_ condition in oral keratinocytes and oral fibroblasts, respectively (Figure 3). In the urea cycles, including 22 metabolites, 11 and two metabolites showed a significant difference in these cells (Figure 4). Time dependency in 2% O_2_ showed less difference comparing with that of 0.5%. As commonly observed features in both cells, lactate, an end product of glycolysis, decreased significantly in 0.5% 72 h (Figure 3a,b). 6-phosphogluconate (6PG) showed a significant decrease, whereas ribose 5-phosphate (R5P), the intermediate metabolites in PPP, significantly increased in the hypoxic oral keratinocytes. A decrease of phosphoribosyl pyrophosphate synthetase1 and 2 (PRPS1 and 2), ribose 5-phosphate isomerase A (RPIA), and ribulose-5-phosphate-3-epimerase (RPE) were observed, which may lead to the accumulation of R5P by producing less PRPP and xylose-5P. Oral fibroblasts showed a decrease of glucose 6-phosphate (G6P) in hypoxia, while oral keratinocytes did not. According to our microarray results, glucose 6-phosphate isomerase (GPI), which is needed to convert G6P to F6P, was higher in hypoxic oral fibroblasts (Table S1). In addition, the NADPH/NADP+ ratio regulates the activity of glucose 6-phosphate dehydrogenase (GPD), which converts G6P to D-Glucono-1,5-lactone 6-phosphate; the latter has a lower tendency in hypoxic oral fibroblasts (data not shown [22]), which may also contribute to the low level of G6P [23]. G6P in oral fibroblasts showed the same tendency with 6PG which indicates the constant metabolism kinetics between them.

Interestingly, oral keratinocytes and fibroblasts showed unique balance changes among intermediate metabolites in the urea cycle and polyamines. Reduced Asn in both hypoxic cells may be because of the decrease of glutamic-oxaloacetic transaminase 1 (GOT1) gene expression level, which generates Asn under hypoxia (Table S1) [24]. Increased 2-oxoglutaric acid (α-ketoglutaric acid; α-KG) was observed in hypoxic oral fibroblasts. Glutamine (Glu), Asn, and fumarate are decreased in both hypoxic cells while ornithine increased in hypoxic oral keratinocytes and did not decrease in hypoxic oral fibroblasts. The decrease of argininosuccinate synthase 1 (ASS1) is consistent with the low level of argininosuccinate in both hypoxic cells (Table S1). Ornithine is catabolized by ornithine decarboxylase 1 (ODC1) to putrescine as a substrate for the synthesis of polyamines, such as spermidine and spermine. As a result of the decrease of ODC1, putrescine and other polyamines (i.e., spermidine, spermine, and acetyl-putrescine) may decrease in contrast to ornithine in both hypoxic cells (Figure 4a,b, Table S1).

## 4. Discussion

Keratinocytes in the stratified squamous epithelium are the major cellular components of the oral mucosa, which are derived from the ectoderm. Fibroblasts in the fibrous connective tissue layer are derived from the mesoderm. Although they are adjacent tissues to the border of the basement membrane, their cellular characteristics vary because of their origin. Since metabolic conditions under the hypoxic microenvironment of their cells are supposed to be further different from the ambient condition, we conducted metabolomic analyses in various oxygen conditions to characterize their metabolomic features.

Oral keratinocytes and fibroblasts under atmospheric oxygen concentration showed the presence of various metabolites with different concentrations. In oral fibroblasts, lysine and ornithine, which are required for collagen synthesis and crosslinking, were higher [25,26]. In contrast, *N*-acetylputrescine, one of the polyamine metabolites important for maintaining epithelial homeostasis, was higher in oral keratinocytes [27]. Thus, metabolites of oral keratinocytes are quite distinct from those of oral fibroblasts even under 20% oxygen concentration.

Metabolomic analyses were conducted under two different environmental factors, exposure time and oxygen concentrations. The cells were exposed for either 24 (short-term) or 72 h (long-term) and then cultured in 2% oxygen, which is the concentration preferred by keratinocyte precursor cells, as well as 0.5% oxygen, which is a severe oxygen concentration, such as in wound healing. Oral keratinocytes showed various metabolic changes in response to hypoxia, especially in glycolysis, the PPP, and the urea cycle. In a temporal metabolomic analysis of the first-line response to oxidative stress, it has been reported that epidermal keratinocytes and dermal fibroblasts showed fluctuation in glycolysis and PPP metabolites within 1 min [28]. Differences in time dependency were little, especially in 2% O_2_, which may be because the time course was excessive in the present study.

In hematopoietic and pluripotent stem cells, PPP was elevated [29,30]. Glycolysis and PPP dominantly produce energy and suppress the generation of ROS, which is an important mediator of cell damage and the cell death process, and it maintains the quiescent state as well. We previously reported that ROS generation was suppressed under hypoxia in oral keratinocytes [14]. Nonetheless, the proliferation of oral keratinocytes increased at 2% and 0.5% oxygen concentrations [14]. A decrease of G6P was seen in hypoxic oral fibroblasts. The upregulation of GPI and downregulation of NADPH/NADP+ that are reported as the hypoxic reaction may retrieve cellular survival and increase proliferation and angiogenesis in hypoxic oral fibroblasts [23,31]. This study revealed that the production of R5P, which is the intermediate metabolite of PPP, increased while 6PG decreased in hypoxic oral keratinocytes. Gene expression changes of 6-phosphogluconate dehydratase (PGD), which produces 6PG from R5P, and transaldolase1 (TALDO1), which produces erythrose 4-phosphate (E4P) and fructose 6-phosphate (F6P) from S7P and glyceraldehyde 3-phosphate (G3P) were inconsistent with their related metabolite changes (Figure 3, Table S1). The activity of PGD and TALDO increases sharply in alkaline pH [32,33], which may lead to low 6PG synthesis or high S7P, since the intracellular environment of hypoxic cells is acidic [34]. 6-phosphofructo-2-kinase/fructose-2,6-biphosphatase 3 (PFKFB3) is an enzyme that switches glycolysis to PPP, and it was elevated in both hypoxic cells (Table S1). It could maintain oral keratinocytes in an undifferentiated state in conjunction with p63, an undifferentiated marker of keratinocytes, and promote collagen synthesis in oral fibroblasts [35,36].

The various metabolites’ concentration in the polyamine pathway and the aspartate to fumarate in the urea cycle were reduced, and the accumulation of ornithine was also found in both hypoxic cell types. The limitation of Asn supports cellular proliferation in hypoxic cancer, which is consistent with our previous study that oral keratinocytes highly proliferate in hypoxia [24,37,38]. Mitochondrial activity is low in hypoxia; as a result, fumarate synthesis decreased following the whole TCA cycle deactivation [39]. A low level of polyamines was observed in both hypoxic cell types. Since polyamines are known to be involved in the keratinocyte cell differentiation process [40], the result in this study was consistent with our previous study that showed that hypoxia maintained an undifferentiated state of oral keratinocytes.

In contrast to oral keratinocytes, oral fibroblasts demonstrated fewer changes in both the PPP and urea cycle. Oral fibroblasts showed the deactivation of overall metabolism, particularly, the metabolites of the TCA cycle, which was lower than those in oral keratinocytes. In addition, both the PPP and the urea cycle in oral fibroblasts are less susceptible to hypoxia than those in oral keratinocytes. In contrast, an increase in α-KG, which is a co-activator of prolyl hydroxylase that is an HIF-1a degrading enzyme, was observed under hypoxia. α-KG, an endogenous intermediate metabolite in the TCA cycle, is a molecule that is involved in multiple metabolic and cellular pathways as well as acting as an antioxidant [41]. Therefore, in oral fibroblasts, α-KG inhibits ROS in hypoxia, which may be involved in cell survival and cellular senescence.

The present study has several limitations. First, we used a limited number of samples because of the usage of primary culture cells. Second, we did not perform any functional assays. By clarifying the molecular response of this metabolic reprogramming, further studies on cellular hypoxic responses are necessary in the future. Focusing on subcellular compartments of glutamine metabolism and its lipogenesis might reveal novel functions [42,43]. Here, we discussed the expression of the metabolic enzymes using only microarray data. Validation of these expressions and activities is required to confirm the reason for metabolomic pathways’ change.

In conclusion, we observed the metabolomic concentrations of oral keratinocytes and fibroblasts for the first time and revealed their holistic changes of two major cell types in the oral mucosa. An increase in PPP and a decrease in polyamine production under hypoxia were detected in this study, which would support our previous data showing that hypoxia can maintain oral keratinocytes in an undifferentiated state and prevent them from cellular senescence. In oral fibroblasts, the overall metabolism changes were smaller than those of oral keratinocytes. Although there were only little changes in any pathway under hypoxia, the concentration of α-KG increased. The metabolic reprogramming findings of our study could contribute to providing insights into stem cell biology, wound healing, and cancer biology.

## Data Availability

The data presented in this study are available on request from the corresponding author. The data are not publicly available.

## Supplementary Materials

The following are available online at https://www.mdpi.com/2077-0383/10/6/1156/s1, Table S1: DNA microarray analysis.
