# Supplementary material for: Metabolomic Alteration of Oral Keratinocytes and Fibroblasts in Hypoxia
Source: J Clin Med. 2021 Mar 10;10(6):1156. doi: 10.3390/jcm10061156 (PMC8001958; doi:10.3390/jcm10061156)
Supplement: Supplementary file 1 [file jcm-10-01156-s001.pdf]

**Supplemental Materials and Methods**

DNA microarray analysis

Whole cell RNA was taken from oral keratinocytes and fibroblasts treated with 2% or 0.5% $O_2$ for 72 hr (N=1). Microarray analysis was performed by Filgen Inc. (Nagoya, Japan) with GeneChip® Human Gene 2.0 ST Array (Thermo Fisher Scientific) or Toray Industries Inc. (Tokyo, Japan) with 3D-Gene Human Oligo chip 25k. The fluorescence images of hybridized microarrays were obtained with GeneChip® scanner 3000 7G (Thermo Fisher Scientific) or 3D-Gene Scanner (Toray). Scanned data were transformed into numerical values and analyzed using Expression Console™ Software (Thermo Fisher Scientific) or 3D-Gene Extraction software (Toray). The method of Robust Multichip Array (RMA) was applied to adjust the data.

**Table S1: DNA microarray analysis**

| Gene Symbol | Gene Description | EC number | Fold Change vs normoxia | | | |
|---|---|---|---|---|---|---|
| | | | Oral Keratinocytes | | Oral Fibroblasts | |
| | | | 2% $O_2$ | 0.5% $O_2$ | 2% $O_2$ | 0.5% $O_2$ |
| ASL | argininosuccinate lyase | 4.3.2.1 | 0.93 | 0.83 | 0.69 | 0.62 |
| ASS1 | argininosuccinate synthase 1 | 6.3.4.5 | 0.57 | 0.61 | 0.20 | 0.80 |
| GOT1 | glutamic-oxaloacetic transaminase 1 | 2.6.1.1 | 0.83 | 0.82 | 0.65 | 0.71 |
| GOT2 | glutamic-oxaloacetic transaminase 2 | 2.6.1.7 | 1.06 | 0.93 | not detected | |
| GPI | glucose 6-phosphate isomerase | 5.3.1.9 | 1.25 | 1.38 | 1.50 | 1.71 |
| ODC1 | ornithine decarboxylase | 4.1.1.17 | 0.49 | 0.49 | 0.16 | 0.17 |
| PFKFB3 | 6-phosphofructo-2-kinase/fructose-2,6-biphosphatase 3 | 2.7.1.105 / 3.1.3.46 | 1.47 | 2.74 | 3.36 | 2.29 |
| PGD | 6-phosphogluconate dehydratase | 4.2.1.12 | 1.04 | 1.11 | 0.86 | 1.09 |
| PRPS1 | phosphoribosyl pyrophosphate synthetase1 | 2.7.6.1 | 0.74 | 0.64 | 0.48 | 0.63 |
| PRPS2 | phosphoribosyl pyrophosphate synthetase2 | 2.7.6.1 | 0.75 | 0.75 | 0.94 | 1.13 |
| RPIA | ribose 5-phosphate isomerase A | 5.3.1.6 | 0.77 | 0.71 | 0.88 | 0.87 |
| TALDO1 | transaldolase1 | 2.2.1.2 | 0.97 | 0.97 | 0.45 | 0.60 |

Gene expression changes of enzymes in PPP and urea cycle (N=1). Relative gene expression of 2% $O_2$ or 0.5% $O_2$ vs normoxia of oral keratinocytes and fibroblasts are shown.
